# Diversity of indoor activities and economic development of neighborhoods

**DOI:** 10.1371/journal.pone.0198441

**Published:** 2018-06-20

**Authors:** Daniele Quercia, Luca Maria Aiello, Rossano Schifanella

**Affiliations:** 1 Nokia Bell Labs, Cambridge, United Kingdom; 2 University of Turin, Turin, Italy; University of Oxford, UNITED KINGDOM

## Abstract

Over the last few decades, public life has taken center stage in urban studies, but that is about to change. At times, indoor activities have been shown to matter more than what is publicly visible (they have been found to be more predictive of future crimes, for example). Until recently, however, data has not been available to study indoor activities at city scale. To that end, we propose a new methodology that relies on tagging information of geo-referenced pictures and unfolds in three main steps. First, we collected and classified a comprehensive set of activity-related words, creating the first dictionary of urban activities. Second, for both London and New York City, we collected geo-referenced Flickr tags and matched them with the words in the dictionary. This step produced both a systematic classification (our activity-related words were best classified in eleven categories) and two city-wide indoor activity maps which, when compared to open data of public amenities and sensory maps of smell and sound matched theoretical expectations. Third, we studied, for the first time, activities happening indoor in relation to neighborhood socio-economic conditions. We found the very same result for both London and New York City. In deprived areas, people focused on any of the activity types (leading to *specialization*), and it did not matter on which one they did so. By contrast, in well-to-do areas, people engaged not in one type of activity but in a variety of them (leading to *diversification*).

## 1 Introduction

What makes urban communities successful? A vital public life, urban studies would suggest [1–3]. To promote that vitality, urban planners act upon the *physical structure* of neighborhoods. In her 1961 book, The Death and Life of Great American Cities, urban sociologist Jane Jacobs argued that city life is a product of the diversity of the physical environment [2]. To promote diversity, four conditions are necessary: 1) neighborhoods must serve more than one function, attracting people at different times of the day and night; 2) city blocks must be small and have dense intersections, encouraging interaction between pedestrians; 3) buildings must be diverse in age and form, supporting a mixture of low- and high-rent tenants; and 4) neighborhoods must have a sufficient density of people and buildings.

Not only structural characteristics but also visual appearance has been theorized to be associated with successful urban communities. Three decades ago, Wilson and Kelling introduced the theory of “broken windows”: visual cues of neighborhood decay (e.g., graffiti, broken windows) lead to disorder and, ultimately, to crime (“one broken window become many”, as they put it [4]). That theory led to a fundamental shift in law enforcement, starting from the city of New York: the focus shifted from apprehending criminals to actively removing visual cues of urban disorder.

It is no coincidence that structural characteristics and visual appearance are both derived from what is publicly visible rather than what goes on in indoor spaces. That is because it is difficult to capture what goes on behind closed doors. However, recently, O’Brien and Sampson did just that [5]. They collected more than a million Boston area 911 dispatches between 2011 and 2012 and used them to create four basic categories of neighborhood social disorder: public disorder, private conflict, private neglect, and public denigration. Based on a temporal analysis, they then examined the causal connections between those factors of social disorder and homicide rates. Contrary to what the theory of “broken windows” suggests, they found that private conflict is by far the strongest predictor for everything else: “increases in public disorder, public violence, guns, and homicides.” For the first time, they showed that what goes on in private is far more telling than what is publicly visible.

Until recently, however, data has not been available to study the impact of indoor activities at city scale. As we shall see in the Related Work section, most of the work has been based on activity diaries, which have limited scale. Indoor activities are simply hard to record. To enrich the methodological toolkit at the disposal of researchers and practitioners, we explored the possibility of using social media data to reliably map activities in indoor spaces. In so doing, we made three main contributions:

- We gathered and classified activity-related words (Section Methods), producing the first urban activity dictionary. We validated this data-driven dictionary by manually inspecting it and by comparing it with previous hand-made peer-reviewed classifications.
- For the cities of London and New York, we collected geo-referenced tags from about 24M Flickr pictures (Section Mapping). Using computer vision, we focus on the tags of those pictures that were taken indoors. We matched those tags with the words in the activity dictionary and produced two city-wide indoor activity maps. We validated these maps by comparing them to other maps which capture sensory and emotion-related descriptors of places and, as such, are good proxies for land use. We found that, in both cities, all the activities overlapped with the urban amenities as well as with sensory and emotional descriptors that one would theoretically expect, adding external validity to our study.
- We then studied the relationship between the socio-economic conditions of neighborhoods and the activities people engage with (Section Neighborhoods and activities). We found that, in deprived communities, people engaged in specific activities (specialization); by contrast, in well-off communities, people engaged with a wide variety of them (diversification). This is the first time that researchers have had the view of indoor activities at city scale and have shown that economic development is associated with not only diversity of social contacts (as Eagle *et al*. showed [6]) but also diversity of indoor activities.

These results open up new opportunities yet come with new challenges as well (Section Discussion).

## 2 Related work

In 1987, Recker *et al*. studied the relationship between activities and people’s mobility [7]. They asked 665 individuals in 249 households to keep travel diaries in which they had to record all trips made. Being a manual process, the classification of activities was coarse-grained, in that, they were classified as being related to work, education, shopping, social, and recreation. A decade later, Janelle *et al*. still used activity diaries but were able to embark in a thorough study on how the use of neighborhoods changed over time [8]. They did so by having their participants manually classify activities based on what-who-where: what type of activities, with whom they were done, and where. In 2004, Kahneman *et al*. published a Science paper that refined the way activities are elicited: they proposed a diary method to gather how study participants spent their time and how they experienced the various activities (e.g., in which mood they were) [9]. In it, activities were richly classified into sixteen types.

Activity diaries have been recently complemented by the use of digital data. To study which places individuals visit at the scale of entire cities, researchers have derived proxies for people’s whereabouts from location-based services such as Foursquare [10, 11] and from mobile phone data [1] [12]. Activities were derived based on what types of places people visited. Again, that allowed for a coarse-grained activity classification, yet it also allowed for the study of important questions in the city context: for example, the study of the relationship between social contacts (who talks to whom) and mobility (where people go) [12] made it possible to answer questions like “do acquaintances visit similar places?”.

All this literature has been about what people do in public (and semi-public spaces) but not in private, with the only exception of O’Brien and Sampson’s work [5]. These researchers analyzed more than a million 911 calls made over two years in Boston, and inferred from them metrics of disorder in both public and private spaces. After a cross-lag longitudinal analysis, they were able to disentangle causal mechanisms: they found that future violent crimes were predicted not by *public* disorder (as opposed to what the broken window theory would suggest [4]) but by *private* disorder (what bubbles up in private spaces). Their work focused on specific types of activities though—those that are socially undesirable.

To sum up, diary approaches are not scalable, activities derived from digital whereabouts or call records (including 911 calls) are coarse-grained or limited. We thus needed a new way of collecting fine-grained activity information at city scale.

## 3 Methods

This work proposes a new way of analyzing indoor spaces from data implicitly generated by social media users. The idea was to search for activity-related words on geo-referenced picture tags. To this end, we needed words and data, both of which are described next.

### 3.1 Gathering social media data

Our first step was to gather social media data against which activity words were then matched. Out of the set of all the public geo-referenced Flickr pictures, we selected a random sample of about 24M public photos taken over a 10-year period (2005-2015) within the bounding boxes of London and New York. For each picture, we collected the anonymized *owner* identifier and the free-text *tags* attached to the photo by the owner. We have chosen to focus on Flickr, rather than on other social media platforms because Flickr content tends to be more geo-salient: geolocated Flickr pictures and their metadata are more likely to be related to the place they have been taken than, for example, geolocated Tweets [13].

### 3.2 Identifying activity words

The second step was to identify a comprehensive list of words reflecting people’s activities.

#### Activity words from Flickr

To collect such a list, we gathered tags people used to annotate their photos. We counted the frequency of tags attached to all our 24M public Flickr photos geolocated in London and New York City, and we manually parsed the 24k unique terms that occurred more than 100 times. A term was then included in the lexicon, if it met at least one of the following requirements. It *i)* was an action verb (e.g., cycling, singing, sing); *ii)* denoted a person who was performing an action (e.g., singer, cyclist); *iii)* was an object that was used to perform mainly one single action (e.g., bicycle); *iv)* was a place that is explicitly dedicated to a specific activity (e.g., concert hall); or *v)* was an acronym that denoted some type of activity (e.g., DIY). We did not include potentially ambiguous words which would be hard to classify (e.g., the word “painting” could refer to the activity of painting or to a museum painting) and generic words that might encompass a variety of activities (e.g., traveling). When an eligible term was encountered, we make sure that, if the term is a verb, we manually include the verb itself and its ‘-ing’ form; for example, if the term “cooking” was found, the term “cook” was also added, even if it rarely occurred. The result of this process yielded a lexicon of 527 terms.

#### Activity words from web documents

Flickr tags might under-represent certain activities simply because those activities do not hold photographic interest. To augment the lexicon and make it more general, we considered three online sources [14–16] that listed indoor and outdoor activities. We manually inspected all these terms following the above procedure on whether to include a term or not, and we obtained 677 unique activity-related descriptors. Unlike in Flickr, most of these descriptors were n-grams (e.g., stamp collecting). As a result, only 12% of this new set overlapped with the words from Flickr. The union of the two sets yielded a lexicon of 1,126 terms.

#### Expansion of activity words

Despite this last addition of terms, the lexicon could still miss some terms. To gauge that possibility, we went back to the Flickr data and, for each seed activity term *t*_*a*_ among the 1,126 in the last set, we computed the probability *P*(*t*_*a*_|*t*_*i*_) for each term *t*_*i*_ that co-occurred with term *t*_*a*_ in at least one photo. A high probability indicated that the term *t*_*i*_ co-occurred almost exclusively with activity *t*_*a*_. We manually inspected the 2k word pairs with probability higher than 0.9. Most of them were existing terms in the lexicon enriched with temporal or spatial information (e.g., “NY marathon 2013”). As a result, we ended up adding only 16 new words to the lexicon that were sufficiently general and, at the same time, we ascertained the good coverage of the lexicon in the previous step.

The result of all the previous steps was the creation of the first activity dictionary, which contains 1,142 English terms. To support future work in the area, the link to the urban dictionary is made publicly available. For the sake of completeness, we will also provide the original lists of words from all the sources we used, before and after every filtering step.

### 3.3 Categorizing activity words

The next step was to give structure to the list of activity words through a systematic classification. To that end, we first built a co-occurrence network whose nodes were activity words, and whose undirected edges were weighted with the number of times the two corresponding words co-occurred as tags in the same pictures. We built this co-occurrence network because the semantic relatedness among words naturally emerges from the network’s *community structure*: semantically related nodes are those that are highly clustered together and weakly connected to the rest of the network. There are literally thousands of different community detection algorithms that have been developed in the last decade [17]. None of them always returns the “best” clustering. However, since Infomap has shown very good performance across several benchmarks [17], we opted for using it to obtain the initial partition of our network [18]. The use of community detection to extract word categories had been successfully tested in previous work that extracted categories of smell and sound words [19, 20]. Compared with other clustering techniques (e.g., LDA [21], K-means [22]), a community detection technique has the advantage of being fully non-parametric and quite resilient to data sparsity.

This partition resulted in many clusters containing semantically-related words, but it also resulted in some clusters that were simply too big to possibly be semantically homogeneous. To further split those clusters, we iteratively applied the community detection algorithm by Blondel *et al*. [23], which has been found to be the second best performing algorithm [17]. This algorithm stopped when no node switch between communities increased the overall modularity. If one were to apply Blondel’s right from the start, the resulting clusters are less coherent than those produced by our approach. The result of those two steps was the grouping of activity words in hierarchical categories.

**Fig 1:**
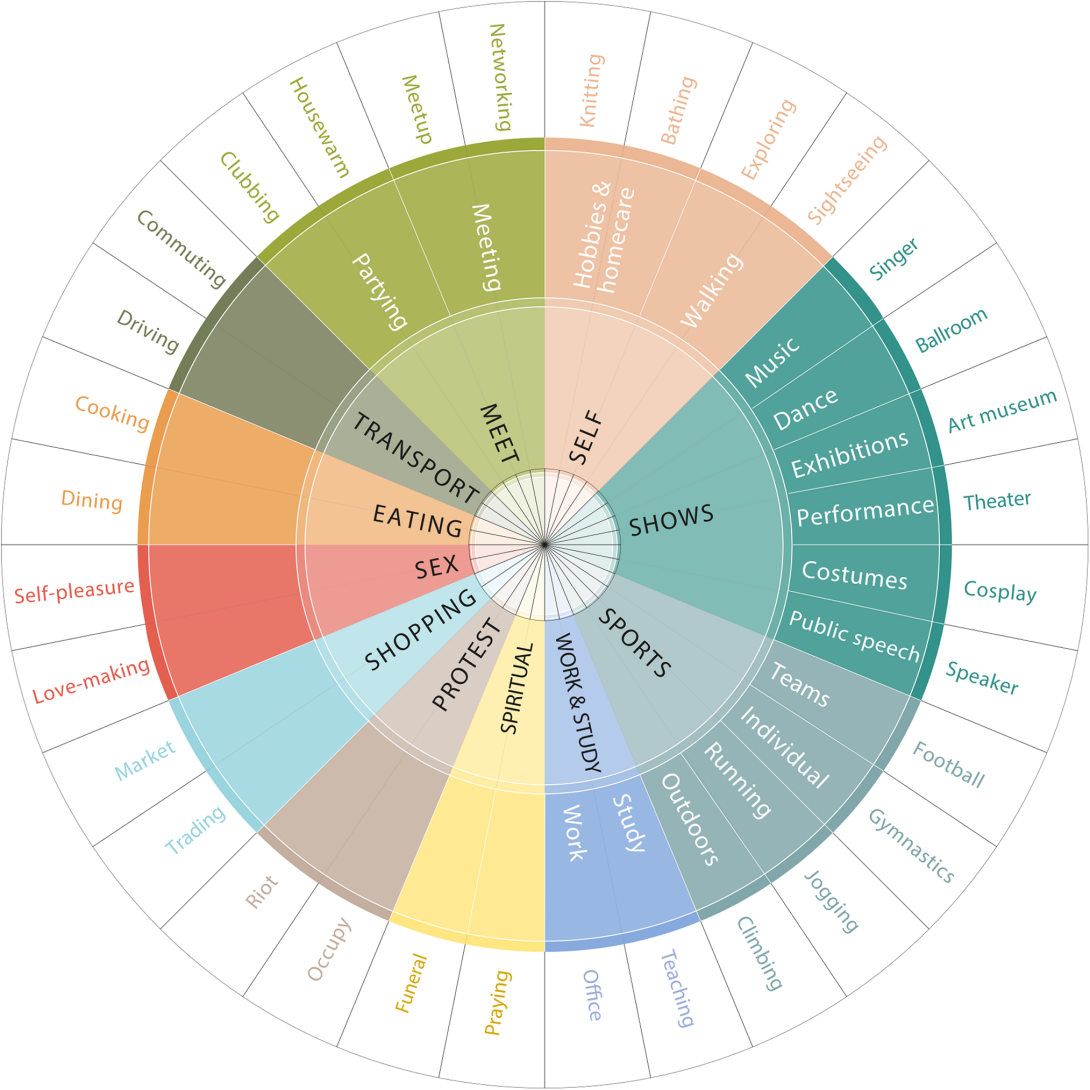
Urban activity taxonomy. Top-level categories are in the inner circle; second-level categories, when available, are in the outer ring; and examples of words are in the outermost ring. Best seen in color.

Fig 1 sketches the resulting classification. It has eleven main categories, each of which has a hierarchical structure with variable depth from 0 to 3.

### 3.4 Identifying indoor activities

We needed to identify which pictures were indoor. To do so, we resorted to Flickr’s vision tags. In addition to user-generated tags, Flickr photos are also marked with *vision tags* [24]. These are tags assigned by a computer vision classifier trained to recognize the type of subject or scene in a photo (e.g., bird, sunset) and whether the scene is *indoor* or *outdoor*. To make sure vision tags successfully identify indoor activities, we searched for photos marked with the tags *indoor* or *outdoor* with confidence level (reflecting accuracy) ≥ 0.9 (respectively, a total of 4*M* and 5.6*M* photos came up), and computed the probability of each term *t* in the lexicon co-occurring with indoor photos ($p_{t}^{in}$) or with outdoor ones ($p_{t}^{out}$) as:

$$p_{t}^{in}=\frac{\#\text{indoor}\;\text{photos}\;\text{marked}\;\text{with}\;\text{tag}\;t}{\#\text{photos}\;\text{marked}\;\text{with}\;\text{tag}\;t}$$ (1)

Table 1 shows the terms ranked by the two probabilities $p_{t}^{in}$ and $p_{t}^{out}$ in descending order. As one expects, the terms reflect predominantly indoor and outdoor activities, suggesting the soundness of the vision approach.

**Table 1:** Typical indoor and outdoor activities derived from tags automatically generated by Flickr’s computer vision algorithms.

Mostly indoor	Mostly outdoor
cooking	airshow
foodspotting	construction worker
home brewing	cycle racing
puzzles	joggers
bath time	kite flying
watching tv	windsurfing
legos	rock climbing
self love	surfing
haircutting	orienteering
partying	sunbathing

### 3.5 Validating the taxonomy

We then performed four main validation steps. First, to validate the dictionary, by visual inspection, we verified that *all* its words were related to urban activities, and that was the case.

Second, to validate the matching between dictionary and picture tags, we matched the geo-referenced Flickr tags with the dictionary words, and found a considerable number of matching tags—i.e., of tags reflecting activities (Table 2).

**Table 2:** Dataset statistics for Flickr photos.

	Users	Items	Activity words	Street segments
London	28,381	454,484	593,602	27,232
New York	8,366	74,381	102,876	14,952

Then, to verify whether those words matched pictures that actually related to activities, we manually checked 100 random Flickr pictures for each top-level category and found that, on average, 87% of the pictures did so. The most accurately matching activities belong to the *eating* category (98%), and the least to *protest* (80%).

Third, to validate the categorization of activity words, we ascertained whether our classification was similar to the hand-made ones produced by researchers in a variety of disciplines. In the field of transport planning, Recker *et al*. asked participants to keep activity diaries and record their activities along the dimensions of work, education, shopping, social&entertainment, and recreation [7]. In the field of behavioral economics, Kahneman *et al*. [9] produced a survey method for characterizing daily life activities and classified them into 13 categories: intimate relations; socializing; relaxing; pray/worship/meditate; eating; exercising; watching TV; shopping; preparing food; on the phone; napping; taking care of my children; computer/e-mail/Internet; housework; working; and commuting. This categorization is strikingly similar to our data-driven one. Among our top-level categories, five of them (meet, sex, shopping, working, spiritual) closely match Kahneman’s. Four of them are more general concepts that possibly include multiple tightly-related categories from Kahneman and expand them (sports includes exercising, transport includes communting, eating includes both the actions of eating and preparing food, and self includes all the activities related to napping, relaxing, watching TV, being on the phone, e-mailing, and doing housework). Furthermore, we find two additional categories (protest and public shows) that were not included by Kahneman. The only activity category we did not capture is taking care of your own children; this category was prominent in Kahneman’s work particularly because his study’s participants were housewives.

## 4 Mapping

### 4.1 Producing indoor activity maps

All the social media data then needed to be mapped onto geographical areas of interest. For London, we used Lower Layer Super Output Areas (LSOAs), which are the smallest UK census areas for which data is available and have a population of around 1,500 inhabitants. For New York, we used census tracts, which have a population of around 4,000 inhabitants. Each indoor photo was assigned to the corresponding geographical area with an additional buffering areas of 15 meters (to account for positing errors of geo-referencing technologies). We then computed the *fraction* of tags in activity category *A* at each census location *l*:

$$f_{A@l}=\frac{\#\text{tags}\;\text{in}\;\text{activity}\;\text{category}\;A\;\text{at}\;\text{location}\;l}{\#\text{tags}\;\text{in}\;\text{any}\;\text{picture}\;\text{taken}\;\text{at}\;\text{location}\;l}$$ (2)

In line with previous work [19, 20], we chose fraction of tags over their sheer volume to avoid any bias given by the high correlation between volume and Flickr penetration rates across neighborhoods.

### 4.2 Validating indoor activity maps

By no means does our dictionary contain an exhaustive list of activities. As such, it was not clear whether we could have observed any relationship between the presence of specific activities in a place and that place’s characteristics. To ascertain whether our geographic units of study made sense, we studied the relationship between sensory maps and activities. We did so with correlation analysis. Since we were dealing with geographic data, for each correlation, we addressed spatial autocorrelation with Clifford *et al*.’s method [25]. This is the tendency for measurements located close to each other to be correlated.

#### Sensory maps

Researchers have recently proposed a new way of collecting olfactory and aural information upon photo tags [19, 20]. They did so by collecting smell-related words and sound-related words (e.g, the researchers conducted “smellwalks” and “soundwalks” in a few cities around the world in which locals were asked to walk around their city, identify distinct odors, and report smell descriptors); matching smell and sound words with social media for the cities of Barcelona and London; and organizing smell and sound words into categories. We borrowed that methodology and repeated it for London and New York City. We then correlated the fraction of each smell and sound category with the fraction *f*_*A*@*l*_ of each activity category at the level of census area. The comparison between activities, sounds and smells is meaningful because the sets of words that describe those three dimensions are largely disjoint. 3% of the unique smell words and 15% of the unique sound words are also activity words. In terms of picture tags volume, 16% of activity tags are also smell tags and 15% are also sound tags.

#### Sensory maps and activities

**Fig 2:**
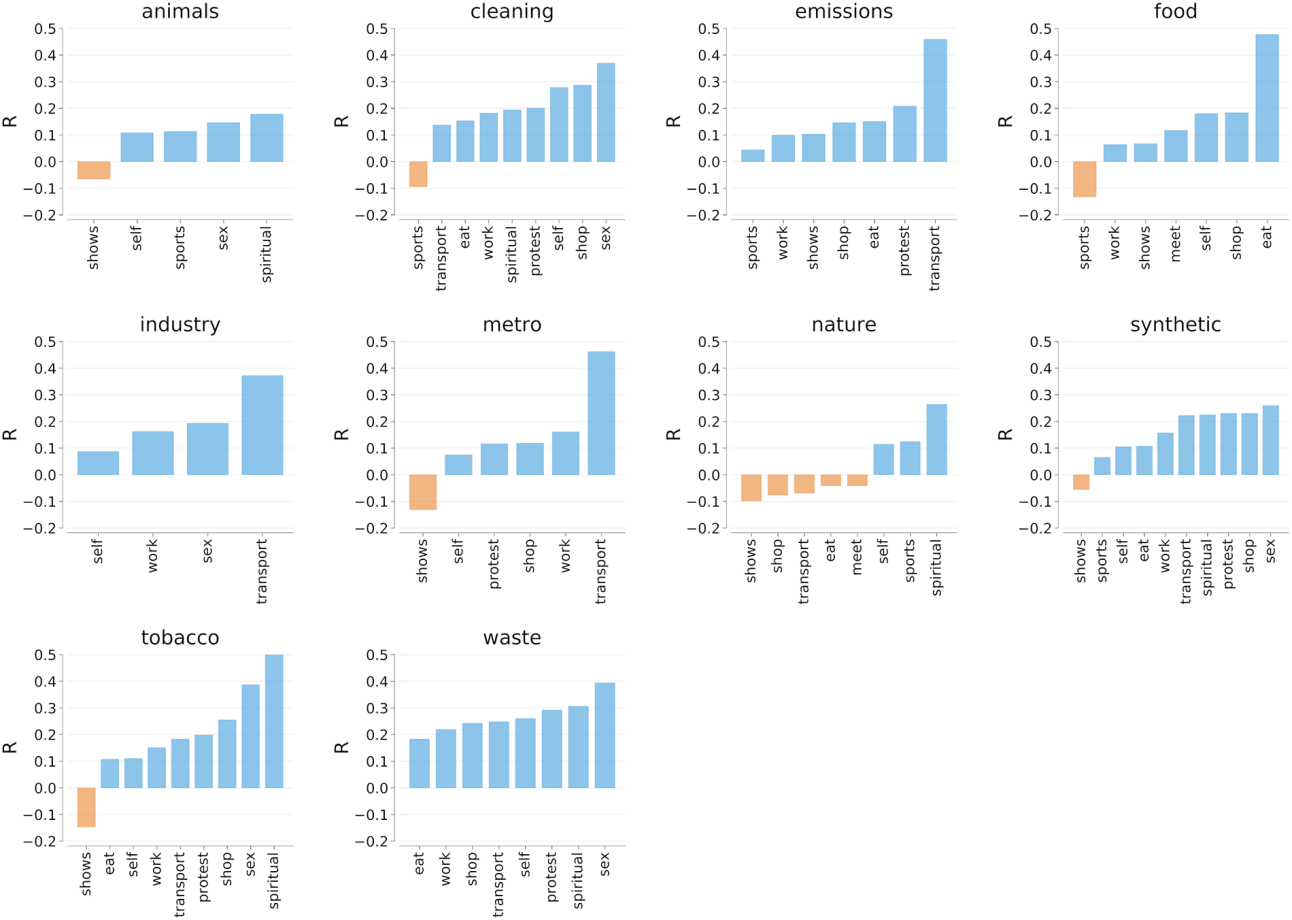
Correlations between the fraction of indoor activities (in the row) and smell categories at the level of census area in London. All correlations are statistically significant with *p* < 0.01.

**Fig 3:**
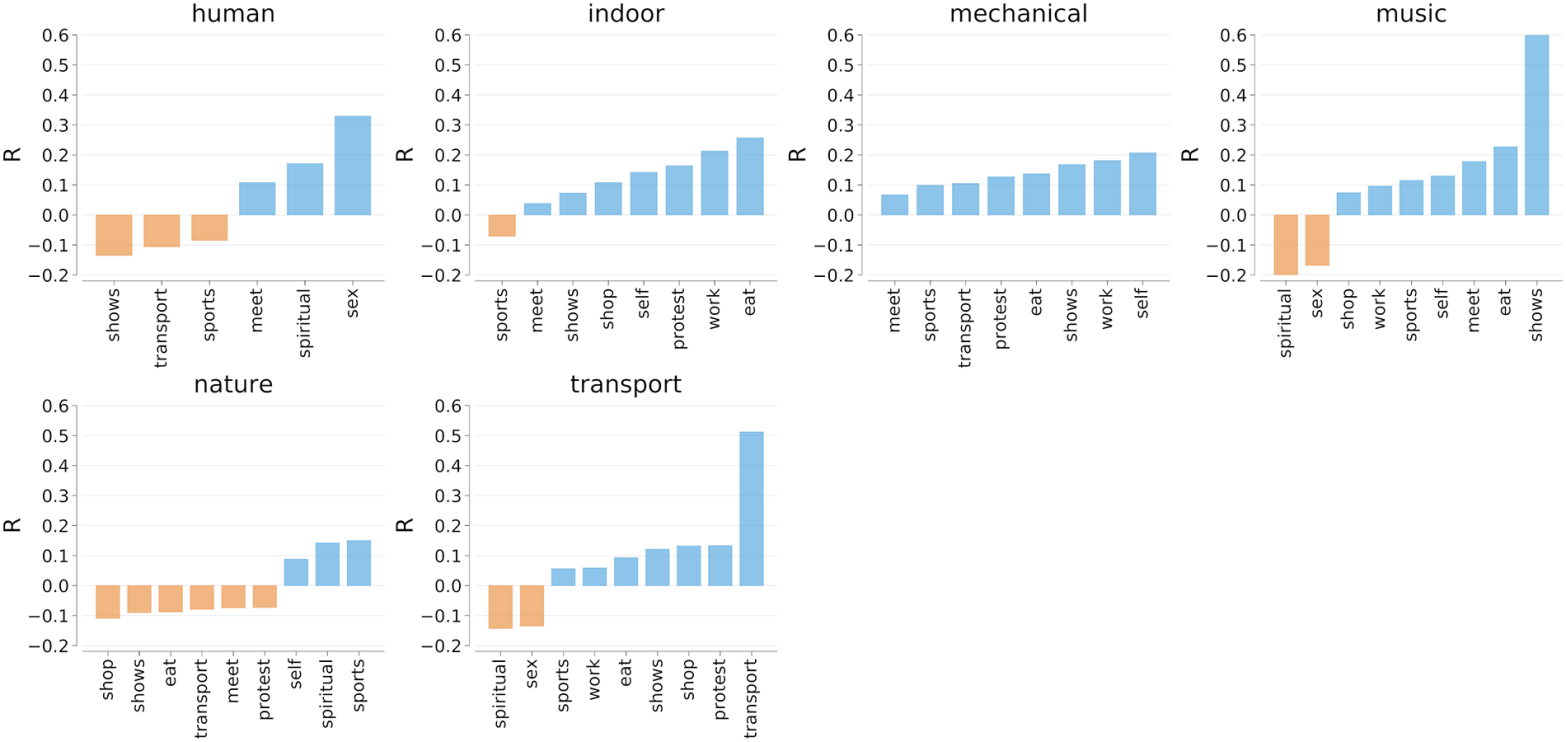
Correlations between the fraction of indoor activities (in the row) and sound categories at the level of census area in London. All correlations are statistically significant with *p* < 0.01.

From the correlations with the smell categories we obtained (Fig 2), we see that eating activities were strongly correlated with the smell of food (*r* = 0.48), and that activities connected to urban transportation (driving, communing) occurred in areas characterized by emission odors (*r* = 0.46), industrial smells (*r* = 0.37) and smell of metro stations (*r* = 0.46). As for the correlations with sound categories, from Fig 3, we see that music was present in places in which people engaged in shows (*r* = 0.61). Sounds produced by people were associated with sexual activities (*r* = 0.33). Urban transportation activities were localized in places characterized by motor sounds (*r* = 0.51) but not by human-generated sounds (*r* = −0.11).

#### Emotions and activities

Looking at a location through the lens of social media makes it is possible to study that location from different points of views. Since activities have a considerable effect on our emotions [9], we set out to study the relationship between activities and emotions. To do so, we used EmoLex word-emotion lexicon [26], which classifies words into eight primary emotions based on Plutchik’s psycho-evolutionary theory [27] (i.e., *anger*, *fear*, *anticipation*, *trust*, *surprise*, *sadness*, *joy*, and *disgust*). Using EmoLex, we computed the fraction of its eight individual emotions at each census location in the two cities:

$$f_{E@l}=\frac{\#\text{tags}\;\text{emotion}\;\text{category}\;E\;\text{at}\;\text{location}\;l}{\#\text{tags}\;\text{in}\;\text{any}\;\text{picture}\;\text{taken}\;\text{at}\;\text{location}\;l}$$ (3)

**Fig 4:**
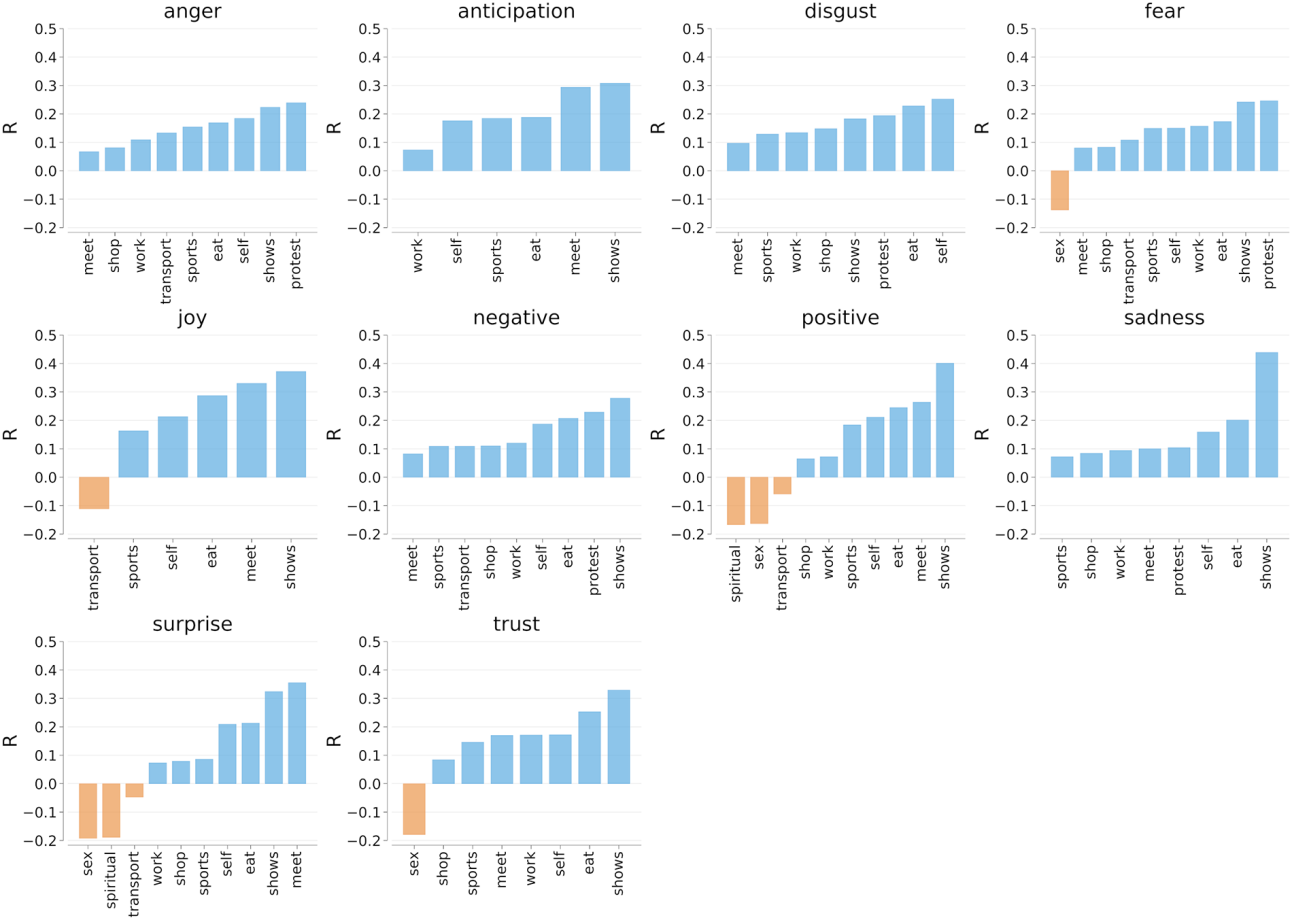
Correlations between the fraction of certain indoor activities in London (in the row) and emotions at the level of census area. All correlations are statistically significant with *p* < 0.01 (e.g., the emotion disgust was not significant).

We then correlated the fraction *f*_*A*@*l*_ of tags in each activity category *A* and the fraction of tags *f*_*E*@*l*_ in each emotion category *E* (Fig 4). We found that joy was mostly associated with going to shows (*r* = 0.37), meeting people (*r* = 0.33), and eating (*r* = 0.29) and tended to be absent in places transportation activities (*r* = −0.11) occurred. Areas that offered opportunities for social gatherings are also associated with surprise (*r* = 0.36)

This comparison involving sensory descriptors and emotion-related words was meant to ascertain the effectiveness of our methodology in mapping indoor activities. We found that work was anti-correlated with joy; music was associated with a variety of situations; eating was associated with joyful emotions and food odors; and transport was associated with anger, traffic emissions, and motor sounds. Those correlations reflected what one theoretically expected, speaking to the external validity of our way of mapping activities.

#### Amenities and activities

Not all activities can take place anywhere; some activities might be more frequent around facilities that might ease them. To add external validity to our analysis, we compare the presence of activities against the presence of physical amenities in the city. To do so, we use data from OpenStreetMap (OSM), a collaborative project to create a free editable map of the world by collecting volunteered geographic information. Points on OSM maps can be annotated with tags of different types. We use the *amenity* tag (https://wiki.openstreetmap.org/wiki/Key:amenity), which captures an assortment of community facilities for visitors and residents alike. After collecting all the amenity tags in both cities, we computed the fraction of the individual amenity types at each census location:

$$f_{O@l}=\frac{\#\text{OSM}\;\text{amenities}\;\text{of}\;\text{type}O\;\text{at}\;\text{location}\;l}{\#\text{OSM}\;\text{amenities}\;\text{at}\;\text{location}\;l}.$$ (4)

**Fig 5:**
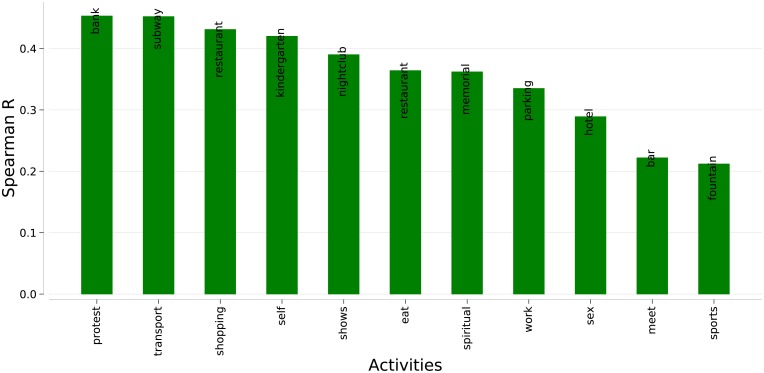
Rank correlations between activities and presence of urban amenities as recorded by Open Street Map. The amenity with the highest correlation for each activity is shown.

The results of the correlation between the fraction of amenities with the fraction of activity types match expectation (Fig 5). Protests were found in financial centers, where bank offices are located (*r* = 0.45). Unsurprisingly, transportation and commuting activities often happens around subway stations (*r* = 0.45) and people were found engaged in work-related activities in areas with abundant parking facilities (*r* = 0.335). Around restaurants, people document online their activity of eating (*r* = 0.36) and shopping (r = 0.43). Spiritual contemplation happens in proximity of memorials (*r* = 0.36). Significant correlations are found between shows and presence of nightclubs (*r* = 0.39), social encounters and bars (*r* = 0.22), sport activities and water fountains (*r* = 0.21). Last, when they share online their sexual activities, people happen to be in areas dense with hotel facilities (*r* = 0.29).

## 5 Neighborhoods and activities

We then studied how the presence of certain activities was related to the neighborhood’s socio-economic conditions.

### 5.1 Socio-economic indicators

The UK Office for National Statistics has published the The Indices of Multiple Deprivation (IMD) every four years since 2000. This is a set of indicators which measure deprivation of small census areas in England known as Lower-layer Super Output Areas [28]. The IMD consists of the following components: Income Deprivation (we used its inverse rank and call it *income*)—number of people claiming income support, child tax credits or asylum; Employment Deprivation (we used its inverse rank *employment*)—number of claimants of jobseeker’s allowance or incapacity benefit; Health Deprivation and Disability (we used its inverse rank *health*)—including a standard measure of premature death, rate of adults suffering mood and anxiety disorders; Education, Skills and Training Deprivation (we used its inverse rank *education*)—education level attainment, proportion of working adults with no qualifications; Barriers to Housing and Services (we used its inverse rank *housing*)—homelessness, overcrowding, distance to essential services; Crime (again, we took its inverse rank)—rates of different kinds of criminal act; and finally a composite measure which is a weighted mean of the other domains (we used its inverse rank *IMD*).

On the other hand, in United States, the Social Vulnerability Index (SVI) uses census data to determine the social vulnerability of every census tract. A number of factors, including poverty, lack of access to transportation, and crowded housing may weaken a community’s ability to prevent human suffering and financial loss in the event of disaster. These factors are known as social vulnerability. For all census tracts in New York City, we collected the following values: Social Vulnerability Index (we used its inverse rank and call it *resilient*); *wealth*—per capita income; *education*—we started from the provided percentage of people with less than 12^*th*^ grade degree and computed its inverse rank; and *housing*—median house value.

### 5.2 Socio-economic analysis

To test the relationship between indoor activities and neighborhood socio-economic conditions, we correlated each socio-economic indicator with each fraction of the 10 activity categories (i.e., *f*_*A*@*l*_). Where necessary, predictor variables underwent a logarithmic transformation. Since all the socio-economic indicators were ranked in a way that higher values were associated with well-to-do conditions, positive correlations uncovered activities in well-to-do areas, while negative correlations uncovered activities in socio-economically deprived ones. We show only the correlations that were statistically significant and were above 0.1, that is, *r* > 0.1 with *p* < 0.01. Again, we used the method by Clifford *et al*. to address spatial autocorrelations [25].

**Fig 6:**
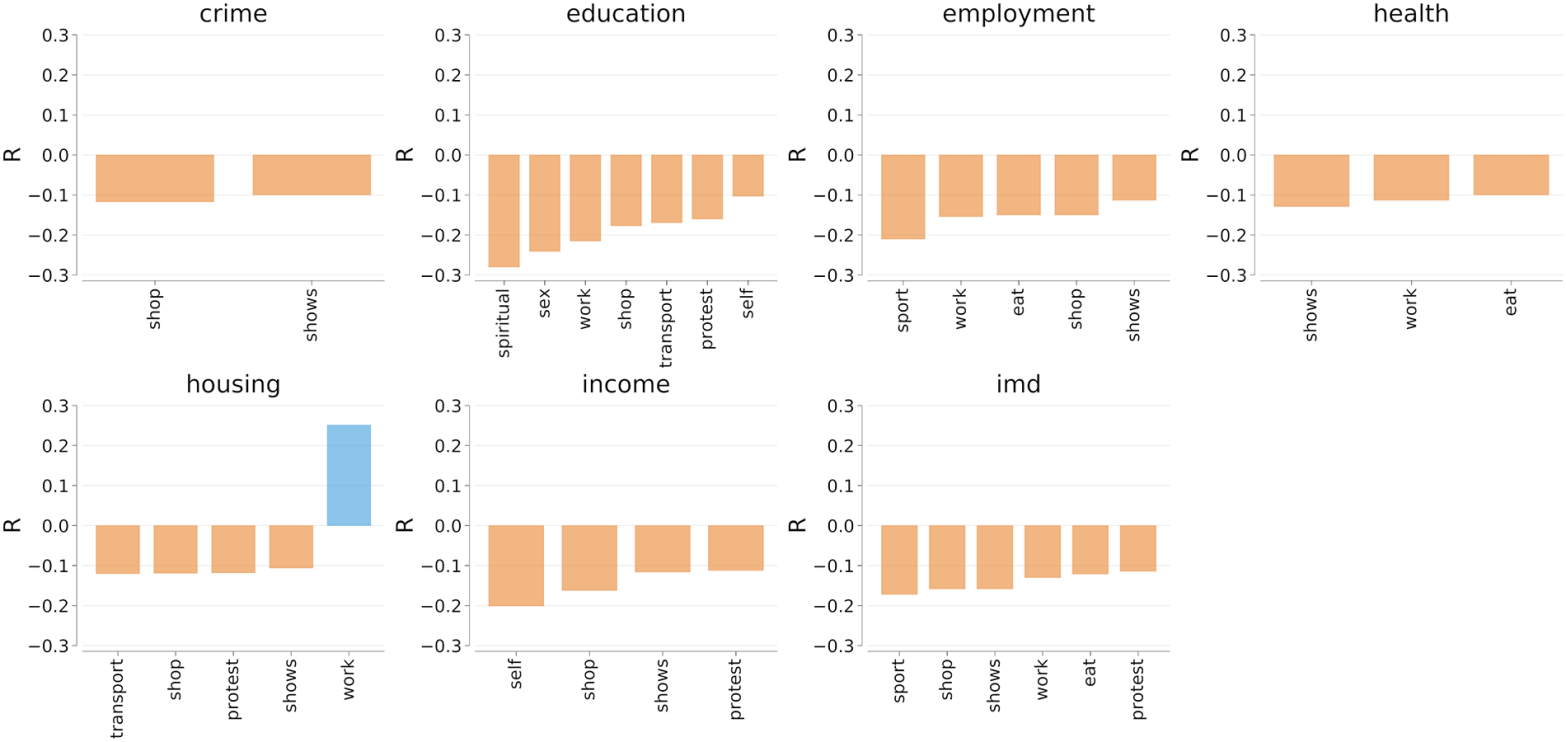
Correlations between the fraction of indoor activities (in the row) and socio-economic conditions at the level of Super Lower Output Area (LSOA) in London. All correlations are statistically significant with *p* < 0.01.

**Fig 7:**
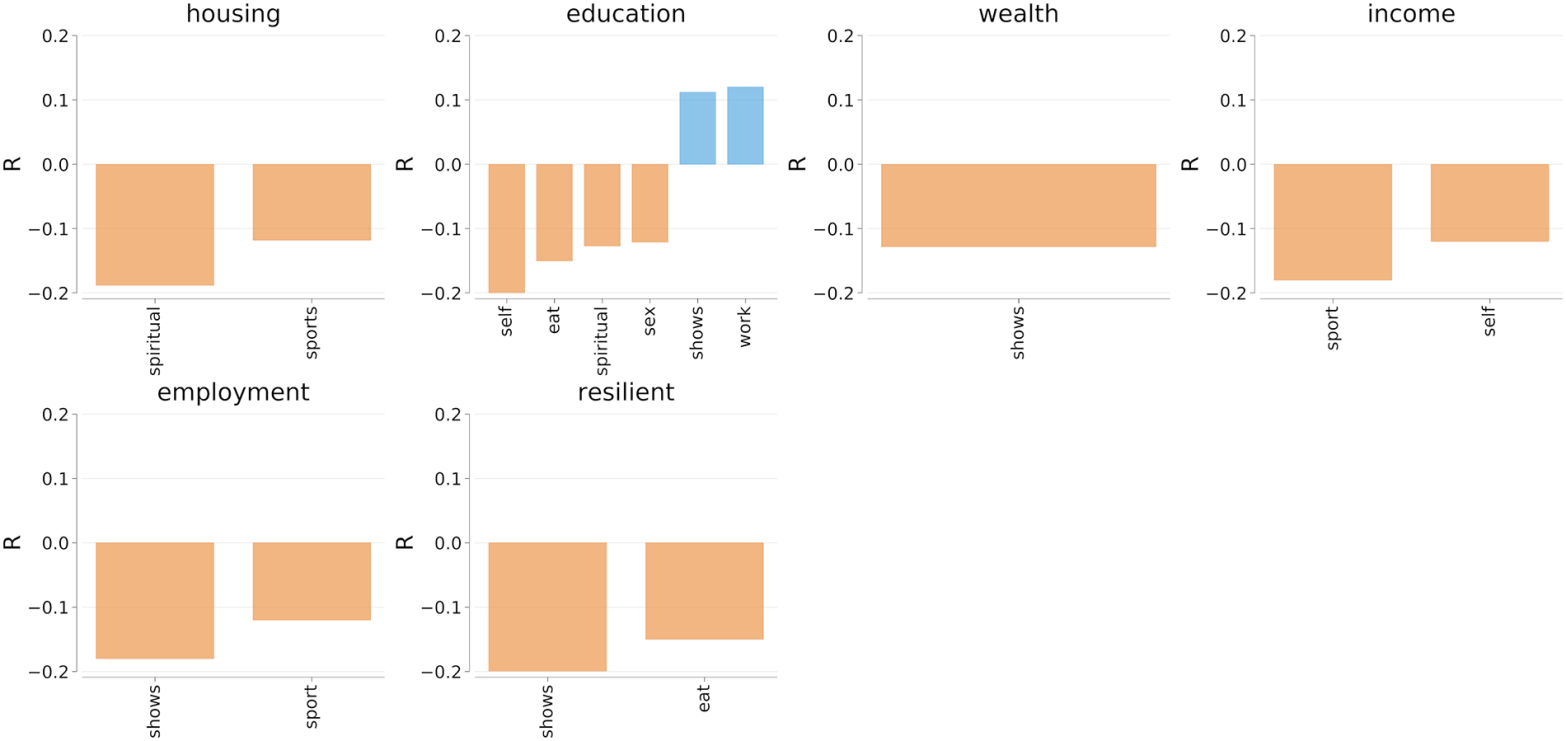
Correlations between the fraction of indoor activities (in the row) and socio-economic conditions at the level of census tract in New York City. All correlations are statistically significant with *p* < 0.01.

By glancing through Figs 6 and 7, one sees that all the activities but one were associated with socio-economically deprived areas. The activity that made exception was *work*, which was mentioned in areas with good access to services in London (*r*(*housing*, *work*) = 0.39) and, together with *shows* with higher levels of education in New York City (*r*(*education*, *work*) = 0.12, *r*(*education*, *shows*) = 0.11).

By contrast, mentions of sport and shows in indoor spaces were associated with areas that were deprived across most of the socio-economic dimensions. That was because, on average, indoor mentions of sport and shows referred to TV content, which was more prevalent in socio-economically deprived areas.

When interpreting these results, one has to consider that our content came from people who posted pictures while managing their profiles and, ultimately, their online identities. So, while doing this work, we assumed the presence of self-reporting bias, that is, we assumed that not all activities were reported, thereby resulting in a non-representative sample of activities. As such, we assumed socially-unacceptable content (e.g., sexually-explicit one) to be rare. However, in our clustering-based classification of activity words (Section 3.3), we already saw that sex took an entire category and, in London, we could even classify picture tags that were geo-referenced in that category. Those individuals lived in areas with low levels of education (*r*(*sex*, *edu*) = −0.20). Activities related to *self*, which reflect hobbies, home care, gaming, and walking, require some free time, so it came at no surprise that people engaging in them were in areas with lower median income in London (*r*(*self*, *income*) = −0.20) and New York City (*r*(*self*, *income*) = −0.20).

Taking all these results together, we learned that most of the activities were associated with socio-economic deprived areas. Therefore, areas that focused on a single activity suffered from socio-economic issues. If specialization does not work, one might wonder whether the opposite—diversification—would. After all, the literature of social networks would support this view. Previous work suggests that the opportunity to interact with a range of more diverse contacts is a proxy for economic growth and well-being [6, 29]. This work is based on the “weak tie” hypothesis: in a social network, individuals with few bridging ties (weak ties) are deprived of information from distant parts of the network and, as such, do not have access to key information to advance their status. Indeed, experimentally, diversity of social contacts has been found to be associated with economic development at both individual level [29] and geographic level [6]. The rationale is that the diversity of contacts results into access to diverse and new ideas, which, in turn, is associated with diverse activities. Therefore, it is reasonable to hypothesize that a community’s economic development is associated with activity diversity. To test that, we computed the activity diversity of location *l* as:

$$\begin{array}{c} D_{l}=1-\sum_{A\in l}f_{A@l}^{2} \end{array}$$ (5)

*D* is low for locations that focus on only one type of activity, while it is high for locations that focus on all activities equally. More generally, the higher *D*, the higher the diversity.

**Fig 8:**
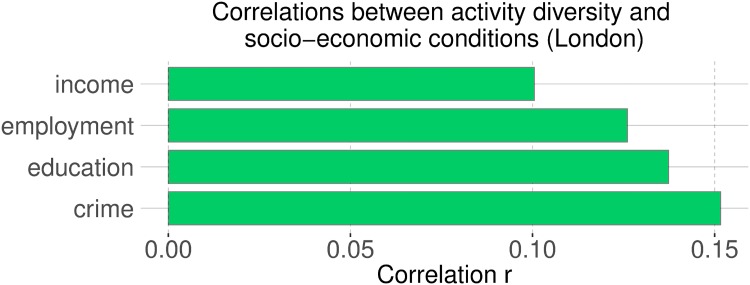
Correlations between activity diversity and socio-economic conditions at the level of of Super Lower Output Area (LSOA) in London. All correlations are statistically significant with *p* < 0.01.

**Fig 9:**
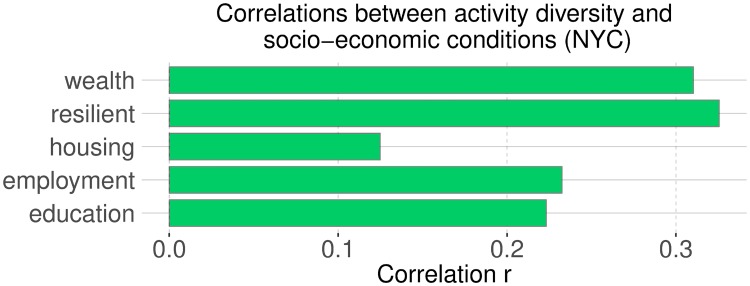
Correlations between activity diversity and socio-economic conditions at the level of of census tract in New York City. All correlations are statistically significant with *p* < 0.01.

**Fig 10:**
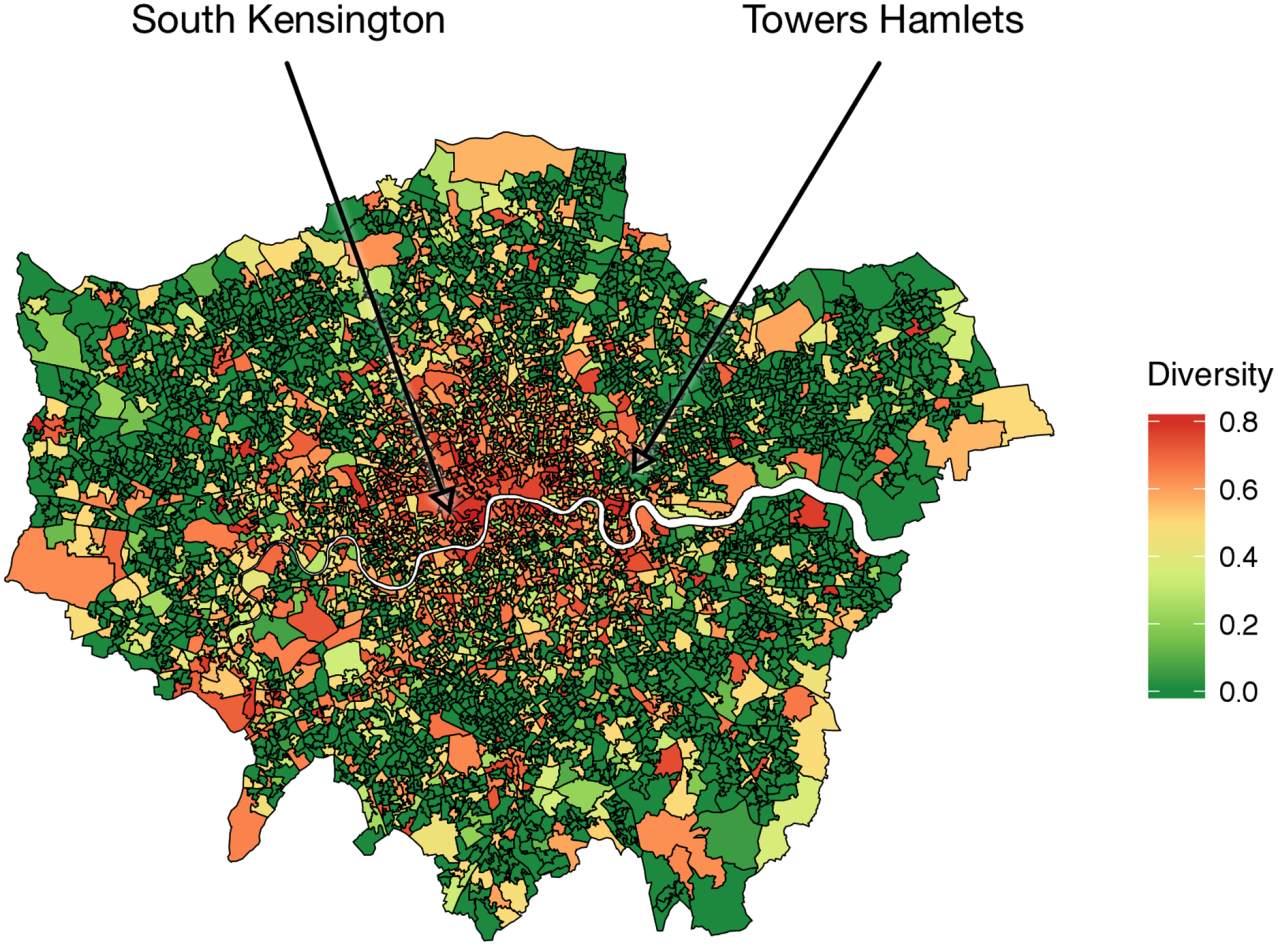
Maps of activity diversity *D* at each LSOA in London.

**Fig 11:**
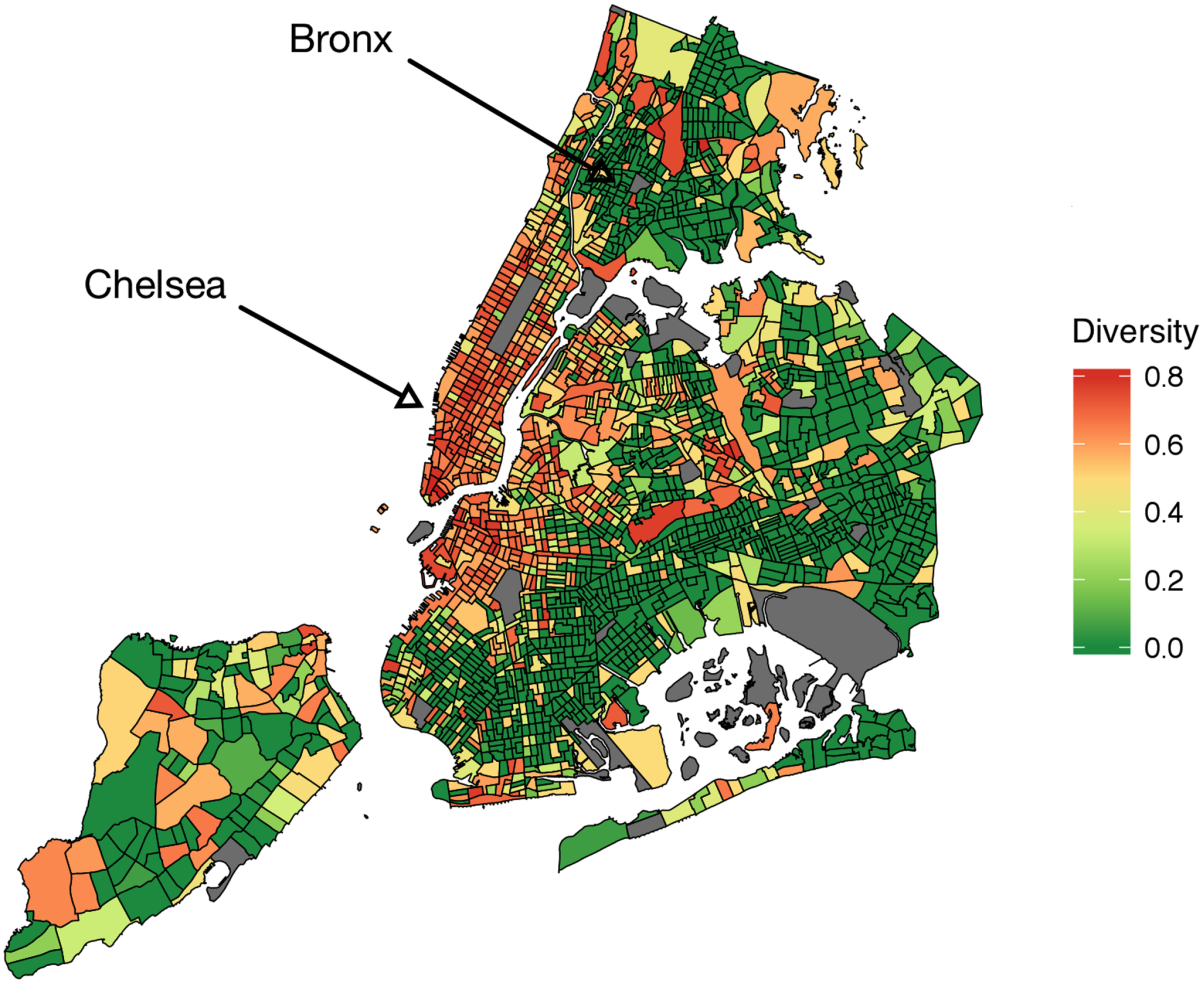
Maps of activity diversity *D* at each census tract in New York city.

We then correlated activity diversity *D* with the socio-economic indicators. As we hypothesized, affluent communities are characterized by activity diversity (Figs 8 and 9). People who engaged in a diverse range of activities tended to live in more affluent communities than those who engaged primarily in one activity. By mapping the diversity (Figs 10 and 11), we see that people of the prosperous South Kensington (London) and Chelsea (New York City) had a balanced activity patterns (high diversity), while people in poorer Tower Hamlets and Bronx (New York City) engaged mostly in a few activities (low diversity).

## 6 Discussion

This work adds to the growing literature exploring the ways in which online data can be used to study the socio-economic development of communities [6, 30]. We demonstrated a significant link between neighborhood deprivation and indoor activities. Next, we discuss some of the limitations of our work and some of the opportunities it opens up.

### Ethical concerns

The metadata we used here existed before but was not in a form usable for profiling indoor spaces. Now we have proposed an effective way of doing so. Unfortunately, this ability penetrates the healthy boundary between the outside world and the indoor. In a similar way, IoT devices (e.g., smart watches) penetrate the boundary between the outside world and the self (e.g., through monitoring of physiological activities). The more the Internet penetrates private spaces (e.g., home, self), the greater the danger of turning mundane activities into data for advertisers and data brokers. To tackle those concerns, research has to go into limiting the data collected by devices and the data accessed by third parties.

### Limitations

The first limitation of our study is the *demographic bias* of Flickr users. The majority of Flickr users are male with a median age of 39. Our results thus represented a subset of all the activities people do. This is one of reasons why we chose London and New York City as they are top-using cities of Flickr. Our analysis is descriptive of the types of activities happening indoors and their correlation with socio-economic indicators, yet we did not propose a *predictive* framework that allows to infer socio-economic conditions from activity data. Similarly, our results do not speak to *causality*. Knowing that a community has a focused activity pattern may be potentially useful for targeting economic development. However, to that end, a cross-lag analysis to potentially observe causal relationships is in order. That would require far bigger sets of data but, in principle, our methodology could still be applied. Having pointed out the limitations of online data, we should also stress that such data shows some advantages over activity diaries, which suffer from two main biases: *a)* sample bias (participants are not representative of the general population); and *b)* response bias (people might perform activities that are different than those they would perform ‘in the wild’—if they were not under study—because of the Hawthorne effect [31]). Online data partly reduces both biases, in that, representative user samples can be extracted (reduced sample bias), and data is captured unobtrusively (with lack of experimental demands, the response bias could be limited).

### Theoretical implications

Our work has three main theoretical implications. First, we showed that Flickr has some connection with the physical reality: the socio-economic characteristics of physical communities have noticeable effects on what users post online. Second, urban researchers have so far focused on public activities; now, based on data unobtrusively captured from social media users, the very same researchers could move forward and study the role that indoor activities play in the urban experience. Third, we have tested the relationship between economic development of neighborhoods and activities people engage with not only at public spaces but also at semi-public or private ones. Previous work has found that individuals benefit from diverse social contacts [29] (e.g., they get access to jobs), and that neighborhoods benefit from diverse (mixed) land use [2] (e.g., they become walkable, and the resulting pedestrian activity promotes vitality). Although these studies suggest that diversity might be beneficial, the relationship between activity diversity and community economic development has never been tested.

#### Practical implications

Our work results into practical applications in a variety of disciplines: *Urban Planning*—planners to date have tended to focus on public activities but we hope that our work might help them re-think their approaches to create stimulating indoor places; *Computer Science*—our methodology allows for the development of new tools for ambiance sensing not necessarily at individual level (because of serious privacy concerns) but also at the level of neighborhood (e.g., after this work, we know that, if a city dashboard could show only one metric among those presented here, that metric would be activity diversity); and *Urban Sociology*—the use of metadata might represent a novel way of studying people’s activities in their physical communities.

## 7 Conclusion

Activities behind closed doors have been out of view of many measurement techniques. This work is the first in examining the role of social media in mapping activities happening in indoor spaces at city scale. Despite self-reporting biases in such data, we still found that, in their private or semi-public spaces, people expressed words of protest and reported sexual activities. More generally, we found evidence that, as opposed to people in deprived communities, people in well-to-do ones tend to diversify their activities. It was not a matter of picture volume or service penetration rates—people in well-to-do communities did allocate their time in a more diverse way. We hope to empower designers, researchers, city managers by offering them a number of methodological tools and practical insights to re-think the role of indoor activities in their work.
